# Examining the contribution of smoking and HPV towards the etiology of oral cavity squamous cell carcinoma using high-throughput sequencing: A prospective observational study

**DOI:** 10.1371/journal.pone.0205406

**Published:** 2018-10-11

**Authors:** Andrew P. Zammit, Rohit Sinha, Caroline L. Cooper, Christopher F. L. Perry, Ian H. Frazer, Zewen K. Tuong

**Affiliations:** 1 The University of Queensland, Faculty of Medicine, Diamantina Institute, Translational Research Institute, Woolloongabba, QLD, Australia; 2 Department of Anatomical Pathology, Pathology Queensland, Princess Alexandra Hospital, Woolloongabba, QLD, Australia; 3 Southside Clinical Unit, Faculty of Medicine, The University of Queensland, St Lucia, QLD, Australia; 4 Department of Otolaryngology, Princess Alexandra Hospital, Woolloongabba, QLD, Australia; Istituto Nazionale Tumori IRCCS Fondazione Pascale, ITALY

## Abstract

Oral cavity Squamous Cell Carcinoma (OCSCC) is a common form of head and neck cancer throughout the developed and developing world. However, the etiology of OCSCC is still unclear.

Here, we explored the extent to which tobacco use, Human Papillomavirus (HPV) infection and genetic and transcriptomic changes contributed to the oncogenesis of OCSCC. In a prospective observational study, we analysed fresh tissue biopsies from 45 OCSCC collected from 51 subjects presenting with OCSCC to the Brisbane Head and Neck Clinics between 2013 and 2015. Exploration of the genetic and transcriptomic landscape of the biopsies were performed using RNA sequencing (RNA-seq) and whole exome sequencing. HPV associated tumours were determined using p16 staining of histological sections and RNA sequencing. Patient demographics including tumor location within the oral cavity, and history of tobacco and alcohol use were correlated with genomic and transcriptomics analyses. About 4.5% of OCSCC were HPV associated. The most frequent mutations in the OCSCC samples were in the *TP53* and *CDKN2A* genes, but no association of specific mutations with HPV or tobacco use was observed. Using weighted gene co-expression network analysis to explore the RNA-seq data, tumors from participants with a history of tobacco use showed a significant trend towards increased mammalian target of Rapamycin (mTOR) signaling and decreased mitochondrial respiration. In conclusion, HPV was shown to be an uncommon association with OCSCC and changes in *TP53* transcriptional regulation, mTOR signaling and mitochondrial function were associated with a history of tobacco use. Larger data sets will be required to enable detection of differences which may help with development of personalized therapeutics in the future.

## Introduction

Head and neck cancers (HNC) are a significant cause of morbidity and mortality in both the developed and developing world [1]. One of the common locations for squamous HNC is the oral cavity (OCSCC). Numerous risk factors have been proposed in the oncogenesis of OCSCC, the most significant being tobacco use. There have been several explanations to the etiology of OCSCC in a non-smoking population, including the human papilloma virus (HPV) and underlying genetic and transcriptomic changes. In particular, the development of OCSCC in a younger, non-smoking population has been of interest to researchers at our institution. Here, we aimed to explore whether HPV, or other underlying genetic and transcriptomic changes, could be responsible for the oncogenesis events in OCSCC. This study was performed utilizing multiple techniques including high-throughput whole exome and transcriptome (RNA-seq) sequencing, which has so far been utilized in a limited number of studies exploring HNC, and examined a population of OCSCCs collected from two Brisbane Head and Neck Clinics between 2013 to 2015.

## Materials and methods

### Participants and sample collection

This project was a prospective observational study. Ethics and governance approval was from the Metro South Human Research Ethics Committee (HREC/12/QPAH/601), the Princess Alexandra Hospital (PAH) Governance Committee and the Royal Brisbane and Women’s Hospital (RBWH) Governance Committee (SSA/12/QPAH/623). A fresh tissue sample and demongraphic information was collected from consenting participants presenting to two Brisbane Head and Neck Clinics (PAH and RBWH) with a squamous cell carcinoma (SCC) localized to the oral cavity (OCSCC). The oral cavity for this study was defined as tumors arising from the anterior tongue, floor of mouth, alveolar ridge, buccal mucosa, hard palate and retromolar trigone. Soft palate and base of tongue lesions were considered to be part of the oropharynx, and were not included in this study. A total of 51 patients were enrolled (37 Male and 14 Female; mean age of 63 years old during time of biopsy (median = 63; lowest = 39; highest = 91). 10 patients reported no alcohol consumption and the remaining declared a rate of 0.25–70 drinks per week (mean = 26.425, median = 21); 7 patients declared that their alcohol consumption was ‘previously heavy’. Lifetime tobacco exposure ranged from 4–124 pack years, with a mean of 34.85 pack years (median = 30). For ex-smokers, the number of years ceased smoking ranged from 2–50 years (mean = 18.61; median = 20). For classification of tobacco use status, 25 were smokers, 18 ex-smokers and 8 non-smokers. Fresh tissue samples (total = 50; only blood was received from 1 participant) were stored initially in RNAlater (Invitrogen, ThermoFisher Scientific) at -80°C. Additionally, a blood sample was obtained from 34 participants, and buffy coat cells were extracted and stored at -80°C.

Formalin fixed, paraffin embedded (FFPE) blocks were available from the pathology services for 27 subjects from one of the two participating hospitals for p16 immunohistochemistry staining. The remaining participants either did not proceed to therapeutic surgery, or had no tissue available for research.

### Immunohistochemistry

Whole FFPE block tissue sections were cut at 3 micrometres onto Trajan glass slides. Staining for p16 immunohistochemistry was performed with p16 antibody (E6H4 clone) (Cintec Roche) on Ventana Ultra with a dilution of 2:1 for 8 minutes, with onboard heat retrieval (CC1) pH8 for 32 minutes and stained with the Ventana Optiview detection kit. p16 was considered positive if there was nuclear and cytoplasmic staining of at least 75% of the tumor cells [2].

### DNA and RNA extraction and library preparation

DNA and RNA were extracted from fresh tissue biopsies and DNA from blood using the Qiagen QIAMP DNA Mini Kit (including an RNAse digestion step) and Qiagen RNeasy Mini Kit, according to manufacturer’s instructions. DNA and RNA were assessed using Nano spectrophotometer, and samples were excluded from testing if the A260/280 was outside of the range of 1.6–2.1. RNA quality was further assessed on the Bioanalyzer (Agilent Technology), and the lowest RIN score was 7.4.

cDNA library preparation from RNA samples was performed using Low Throughput Sample Protocol from Illumina TruSeq RNA Sample Preparation Kit v2 (catalogue #15027387, #15025063 and #15027084 obtained from Illumina, San Diego, CA, USA). Briefly, mRNA was purified from total RNA using Ampure magnetic beads with attached poly-T oligos. Purified mRNA was fragmented with divalent cations and high temperature. Fragmented mRNA was mixed with random primers and reverse transcriptase and amplified through one PCR cycle (lid at 100°C, 25°C for 10 min, 42°C for 50 min, 70°C for 15 min, hold at 4°C) to create first strand cDNA. First strand cDNA fragments were then incubated with DNA polymerase I at 16°C for 1 h to synthesize second strand cDNA. Resultant double-stranded cDNA fragments were end repaired (3’ overhang was cleaved and 5’overhang was filled with A to create blunt ends), and ligated to adapters (kit contains 24 different adapters). The products were purified and amplified by PCR (lid at 100°C, 98°C at 30 s, 10 cycles of: 98°C for 10 s, 60°C for 10 s, 72°C for 10 s; 72°C for 5 min, hold at 10°C) to obtain the final cDNA library. Quality and concentration of cDNA libraries were checked using Agilent 2100 Bioanalyzer and DNA 1000 LabChip kit according to manufacturer's protocol.

Of the 50 fresh tumor tissues collected, 45 whole exome sequencing libraries were successfully generated (from 45 tumor tissue samples) and 41 cDNA libraries were generated for RNA-seq (from 38 tumor tissue samples; duplicate cDNA libraries were generated for 3 randomly selected samples to control for batch effects associated with sequencing on separate occasions). In addition, 34 DNA libraries were generated for whole exome sequencing of blood from 34 blood samples. For analysis of the whole exome sequencing data, we were able to match 25 blood samples with corresponding tumor tissues from the same patient; sample information for 2 blood samples were mismatched, and whole exome sequencing was not performed on tumor tissues matching to the remaining 5 blood samples. Whole exome sequencing data for all 34 blood samples were used to generate the panel of normal for variant calling (see below). Of the 45 tumor tissue samples processed for whole exome sequencing, 34 tumor tissue samples had both whole exome sequencing and RNA-seq libraries generated. The remaining 11 tumor tissue samples were only processed for whole exome sequencing.

### Sequencing

Whole exome sequencing was completed at the Australian Genome Research Facility (AGRF). Samples were sequenced on the Illumina HiSeq 2500 platform with 2x100 bp paired-end reads using the Agilent SureSelect Human All Exon V6 capture/enrichment system. RNA sequencing was completed at The University of Queensland Centre of Clinical Genomics. Samples were sequenced on the Illumina HiSeq 4000 platform with 2x100 bp paired-end reads. Description of bioinformatics tools and workflows used to analyze sequencing data are found in the eMethods. The sequencing data described in this study is deposited in ArrayExpress accession E-MTAB-6448.

### Bioinformatics pre-processing

Somatic variant discovery on whole exome sequencing data was performed after preprocessing steps as per outlined in the Genome Analysis Tool Kit (GATK) Best Practices workflow for whole exome sequence [3], including mapping of reads to hg19 reference genome using BWA-MEM [4], reordering, sorting and marking PCR duplicates using Picard (Broad Institute), base recalibration, insertion and deletion (Indel) realignment and contamination estimation using GATK scripts.

RNA-seq fastq files was assessed for quality control using the fastQC tool [5]. RNA read pairs were mapped to the GENCODE v25 human reference genome (GRCh38.p7) [6] using the RNA sequencing STAR aligner [7]. STAR RNA sequencing read alignment was also performed using a combined genome of human and HPV16 (NC_001526.4) to map reads that correspond to HPV16 genes. Raw read counts for genes were obtained by counting reads using htseq-count [8].

### Whole exome sequencing variant calling and other bioinformatics tools

Three well-known somatic variant callers were used in this study: MuTect (v1.1.7) [9], MuTect2 (GATK 3.7, still in beta) and VarScan2 (v2.4.3) [10]. MuTect was used to call for somatic short nucleotide polymorphisms (SNPs) while MuTect2 and VarScan2 were used to simultaneously call both SNPs and Indels in matched tumor-normal pairs. A panel of normal was created using MuTect and MuTect2 in artifact detection mode using whole exome sequence data from the blood samples acquired from 34 patients recruited in this study. This panel of normal was used as another blacklist for common germline mutations found in this cohort for MuTect and MuTect2 workflows. Variant filtering for VarScan2 output was performed as per described in [11]. In all three variant calling workflows, common germline mutations found in dbSNPv140 database were removed while known somatic mutations found in the Catalogue of Somatic Mutations in Cancer (COSMIC) v80 database were retained (MuTect and MuTect2 –part of input during calling process; VarScan2 –mutations were annotated and filtered against the 2 databases using SnpSift [12]. Filtered variants were then annotated using Oncotator version 1.8.0.0 [13]. The MutSigCVv1.3.01 [14] computational method hosted on the Broad Institute’s GenePattern public server was used to identify genes that are significantly mutated. Filtered outputs were visualized using the Integrative Genomics Viewer [15, 16] to verify if variants were inappropriately called. The summarized mutation plots were constructed using GenVisR [17] and maftools [18] R packages from BioConductor.

Copy number variants were called using VarScan2’s somatic copy number alteration calling workflow using matched tumor-normal pairs. Circular binary segmentation was performed using the DNAcopy library from BioConductor [19]. Beta allele frequencies at germline heterozygous loci in tumor samples from MuTect output and copy number alterations identified from VarScan2 were used as input for predicting tumor and subclonal purity and ploidy using the BubbleTree visualization tool from BioConductor [20].

### Differential gene expression testing

Raw read counts per gene from the RNA-seq experiments were normalized using the LIMMA-voom tool in R, which applies linear modelling to voom-transformed read counts [21]. Differential gene expression was performed using the empirical Bayes statistics testing method embedded within LIMMA. Fold-change of > 1.25 or < -1.25 and Benjamini-Hochberg adjusted p-value < 0.05 was use as a cut-off threshold to assess for significant differential gene expression.

### Weighted Gene co-expression network analysis (WGCNA) and pathway analysis

Voom-transformed expression data from the RNA sequencing experiments were used as input and a weighted gene network was generated using the WGCNA package [22]. Strongly correlated gene-gene pairs are identified and grouped into distinct colored modules based on a scale-free topology criterion where connectivity of genes follows a power-law distribution, highlighting strongly connected gene-gene pairs while penalizing weakly connected pairs. The summary profile (eigengene) of each module was correlated with external clinical traits and looked for the most significant associations. To facilitate biological interpretation, Gene Ontology functional enrichment analysis was performed for the genes contained in the various modules using the Bioconductor packages within R as well as using gene set over representation analysis with software available on the ConsensusPathDB tool website [23]. Specifically, genes from modules of interests from WGCNA analysis were extracted and analyzed with ConsensusPathDB to assign the most overrepresented biological pathways that are enriched by the input list of genes.

## Results

### HPV detected in limited subset of OCSCC Patients

HPV testing is routinely performed for oropharyngeal HNC (OPSCC). HPV positivity in OPSCC is typically assessed using p16 histological staining, as accumulation of p16 protein is a surrogate marker for active HPV E7 oncoprotein activity [24]. However, the reliability of p16 staining in OCSCC is uncertain [25, 26], and this test is not performed routinely. Of 33 OCSCC tested for HPV mRNA in the current study, 2 were positive (both HPV16), while 31 were negative (S1 Fig). Immunohistochemical staining for p16 positivity was performed with a positive result if > = 75% of tumor cells were positive (S2 Fig), based on published methods for scoring oropharyngeal SCC [2]. Of the 33 subjects tested for HPV DNA, results for p16 immunohistochemical staining were available for 27. Of these, 6 were p16 positive, and one of these was positive for HPV mRNA, while 21 were p16 negative. A furthersubject positive for HPV16 mRNA was not tested for p16. The general prevalence and lack of HPV in OCSCC is consistent with other published results (~5–6%) [27, 28].

### TP53 and CDKN2A are the most frequently mutated genes in OCSCC patients

**Fig 1:**
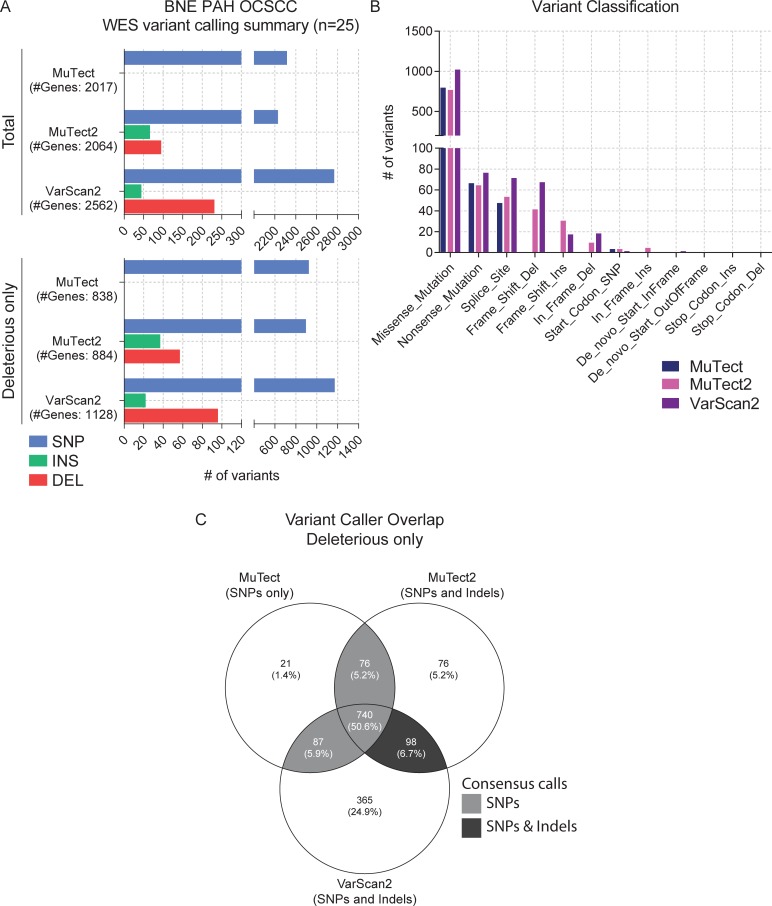
Tumor DNA mutation frequency in Oral Cavity Squamous Cell Carcinomas. DNA was sequenced from 25 subjects with OCSCC, using paired tumor and normal DNA samples, and sequence data was examined for tumor specific mutations using MuTect, MuTect2 and VarScan2 (A) Summary of total and potentially deleterious somatic short nucleotide variations. (B) Frequency of potential deleterious somatic mutations by variant classification. (C) Consistency between the three variant calling programs in calling of single nucleotide variants and indels.

**Fig 2:**
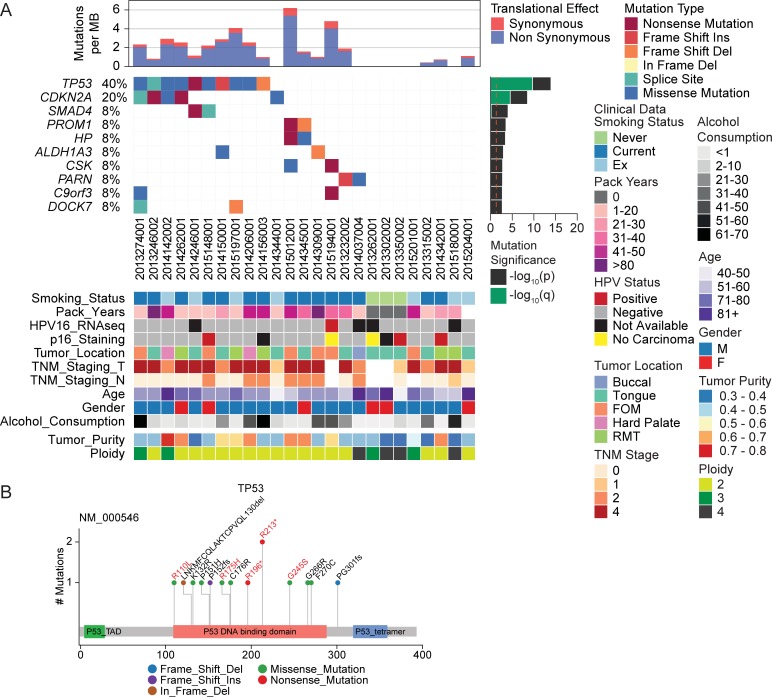
Analysis of OCSCC somatic mutations. Somatic mutations were determined using paired OCSCC tumor/somatic DNA samples (n = 25), and defined as significant if called as non-synonymous by at least two variant calling software programs. (A) Waterfall plot of significant mutations, with the most deleterious mutation for each gene organized hierarchically, and showing mutually exclusive or co-occurring events. Mutations are ranked accordingly to significance assigned by MutSigCV analysis. Clinical traits are matched to the samples and displayed as a heat map. Tumor purity and ploidy values predicted from BubbleTree are included. (B) Lollipop plot of somatic mutations identified in TP53. Previously reported variants are highlighted in red.

Tobacco usage, alcohol consumption and HPV infection have been identified as risk factors for OCSCC and OPSCC [28, 29]. To examine whether these risk factors would be associated with an increased frequency of non-conservative somatic gene mutations, three somatic variant callers (MuTect [9], MuTect2 and VarScan2 [10]) were used to compare whole exome DNA sequencing data from 25 tumor samples from the current study with that from matched normal tissue. The three pipelines predicted ~40% of identified somatic mutations in an average of 950 genes across the three pipeline as non-conservative (MuTect: 924/2315, 39.99%; MuTect2: 990/2390, 41.42%; VarScan2: 1290/3049, 42.23%) (Fig 1A). As expected, missense and nonsense mutations formed the majority of non-conservative mutations. Splice site mutations and frame shift/in frame Indels were the next most common non-conservative mutations (Fig 1B). As there was only a ~50–55% overlap in predicted mutations between the three pipelines (Fig 1B), calls that were present in at least two pipelines were used to define a conservative list of somatic variants. Using this list, the most common mutations observed were in *TP53* (40%) and *CDKN2A* (20%) [30] (Fig 2A). Summarized mutation plots for individual callers are available in S3 Fig. The mutations found in *TP53* were mostly described mutations in the DNA binding domain [31] (Fig 2B). We did not identify any non-conservative mutations in *TP53* and *CDKN2A* in tumor tissues from never-smokers (Fig 2A). Furthermore, *TP53* and *CDKN2A* mutations were not seen in the HPV16 mRNA positive tumor (#2015194001, current smoker), or for two of the three samples that were found to stain positive with p16 immunohistochemistry, but HPV mRNA negative (#2013350002, never smoker; #2014342001, current smoker). Mutations in *TP53* was found in OCSCC tissues from 46% of current-smokers and 42% of ex-smokers, while *CDKN2A* mutations were found in OCSCC tissues from 20% of current-smokers and 28% of ex-smokers. No association was observed between the mutation profile and any measured clinical covariates, including age, gender, alcohol consumption, tumor location, tumor staging and predicted tumor purity (Fig 2A).

To gain a perspective of the subclonal architecture of the OCSCC tumors in this study, the recently published BubbleTree [20] prediction analysis was applied to the tumor DNA dataset (S4–S6 Figs). The analysis uses tumor beta allele frequencies at germline heterozygous loci and somatic copy number alterations to predict the prevalence of (sub)tumors. Overall, tumor purity ranged from ~25%-80% and the median was 43% (Fig 2A). Samples with predicted low tumor purity also generally displayed low frequency of somatic mutations. Eight of the 25 samples were predicted to be polyploid (Fig 2A). The analysis also predicted presence of subclonal events in 19/25 samples, confirming that OCSCC tumors display high degree inter-patient heterogeneity [32].

Overall the results from our whole exome sequencing study were consistent with previous work for OCSCCs, identifying that mutations in *TP53* and *CDKN2A* were the most prevalent in our cohort of OCSCC primary tumors. However, we did not detect the presence of somatic mutations in other genes previously associated with OCSCC such as NOTCH1, KRAS and PTEN.

### Clinical covariates are associated with transcriptomic changes in OCSCC

**Fig 3:**
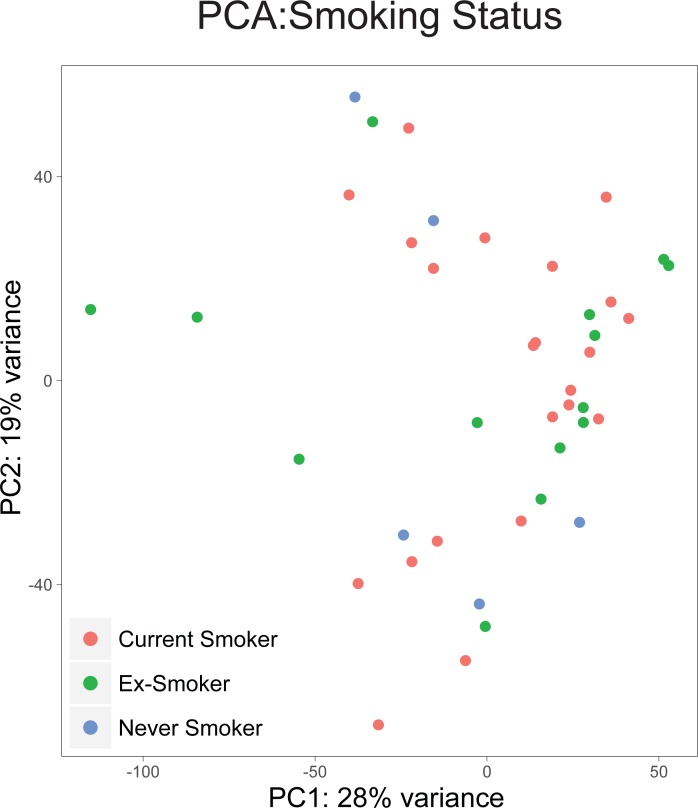
Analysis of tumor mRNA from OCSCC according to smoking history. Principal component analysis of mRNA expression levels determined in OCSCC (n = 41) by RNA-seq. Samples are color-coded by their smoking status.

To determine whether gene expression in the primary OCSCC tumors varied according to clinical and demographic data, a principal component analysis of tumor mRNA data was performed, using the top 500 varying genes from 41 primary tumors. No clustering was observed according to recorded smoking status (Fig 3), and there were no genes that attained statistical significance when differential gene expression testing was performed between the three smoking status groups (S7 Fig).

**Fig 4:**
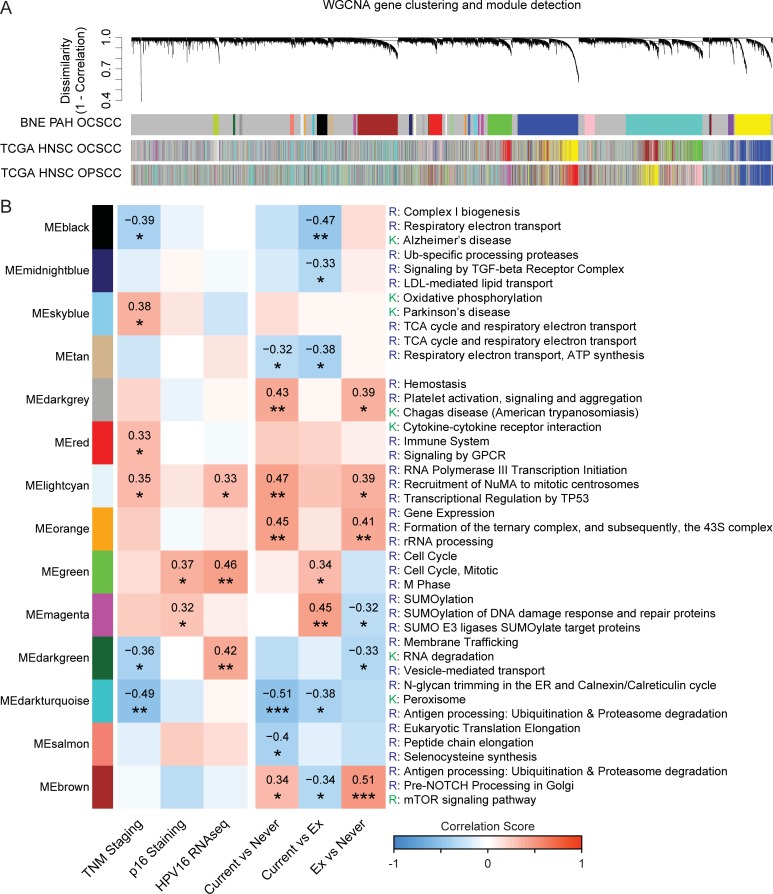
Weighted gene co-expression network analysis of RNA-seq data from OCSCC. (A) Gene dendrogram and gene modules detection in OCSCC samples (n = 41) from the current study, compared with TCGA oral cavity (n = 339) and oropharynx (n = 74) data sets. Gene networks were constructed separately and detected modules in each network were plotted under the current study OCSCC gene dendrogram, to qualitatively assess for preservation of gene modules. (B) Module-clinical trait correlation table. Modules that displayed significant correlation patterns with measured clinical traits are plotted. Top 3 KEGG or Reactome pathways enriched in each module are listed on the right.

To explore co-variance not reflected in the principal component analysis or differential gene expression testing, a Weighted Gene Co-Expression Network Analysis (WGCNA) approach was used to construct a gene network and to seek correlations with clinical covariates. To validate the RNA-seq data from the Brisbane study, this was compared with data from the TCGA HNSC dataset (n = 528 subjects). We constructed separate gene networks for samples that were categorized as oral cavity-related (n = 339) or oropharynx-related (n = 74) and overlaid the gene modules information with the Brisbane OCSCC cohort. The gene modules were relatively well preserved in each cohort (Fig 4A). A consensus network was then constructed between the Brisbane study and the TCGA oral cavity cohort. High network preservation was observed in the consensus gene network, but there was little agreement with the clinical trait correlation patterns (S8 Fig). The only exception was in dark green module (33 genes) and the top gene ontology pathways enriched by the list of genes include “translational initiation” and “regulation of gene expression”. The lack of observed correlation to clinical traits likely reflects a highly heterogeneous cohort of samples within the TCGA HNSC oral cavity subjects.

**Fig 5:**
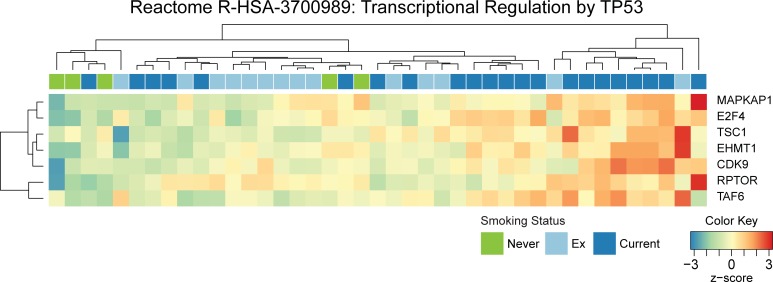
TP53 related gene expression correlations with reported smoking history in OCSCC patients. (A) Normalized gene expression values for each gene from the Light Cyan module from Fig 4 are expressed as a relative z-score and plotted as a heat map. Position of samples and genes are distributed according to a hierarchical sort. Clinical information on smoking status is included on the top of the heat map.

Analysis restricted to the gene network constructed within the Brisbane cohort was then performed and showed significant correlation between several modules and clinical traits (Fig 4B). Advanced TNM staging showed both positive and negative correlation with genes changes related to oxidative phosphorylation (sky blue and black respectively), and positive correlation with immune response (red), RNA transcription and TP53 transcriptional regulation (light cyan); p16 staining was positively correlated with cell cycle regulation (green) and SUMOylation (magenta), including SUMOylation of DNA damage response and repair proteins; HPV16 (RNA-seq) positive samples were positively correlated with cell cycle (green), TP53 function and transcription regulation (light cyan) and vesicle trafficking (dark green) related functions; current smoking was negatively correlated with gene changes associated with mitochondrial respiration (black and tan), TGF-beta signaling (midnight blue), and antigen processing and presentation (dark turquoise); current and ex-smoking positively correlated with TP53 function (light cyan), platelet activation and signaling (dark grey), antigen processing and mammalian target of rapamycin (mTOR) signaling (brown) (Fig 4B), and increased expression of genes encoding *MAPKAP1*, *E2F4*, *TSC1*, *EHMT1*, *CDK9*, *RPTOR*, *TAF6* (Fig 5). *MAPKAP1*, *TSC1* and *RPTOR* are also involved in mTOR signaling, which has been associated with tumor growth and immune regulation in the local microenvironment of oral cancers [33].

In conclusion, our data suggest that HPV and smoking status do not correlate with specific somatic mutations in OCSCC, and that in patients with history of smoking and/or HPV positivity, gene changes downstream of TP53 coincide with mTOR-related gene expression changes.

## Discussion

This study examined possible etiologies of OCSCC, particularly the contribution of HPV and smoking. Overall it appears that HPV plays a minimal role in OCSCC carcinogenesis. This is in contrast to OPSCC, where non-smoking patients typically have disease which is the result of HPV infection [34, 35]. This work was reflected in the recently published TCGA data with RNA-seq, which showed only 6% of non-oropharyngeal head and neck cancer had high HPV reads and increased *E6*/*E7* expression [36]. This has clinical significance in terms of future trends of OCSCC incidence; whilst OPSCC incidence is expected to decline following HPV immunization, future incidence of OCSCC will not be affected by HPV immunization. Additionally, screening programs for HNC utilizing HPV presence will be unlikely to detect significant numbers of OCSCC, thus necessitating other screening programs for cancers within the oral cavity.

The most commonly mutated genes were TP53 and CDKN2A. TP53 has been shown to be a key driver of oncogenesis in a large numbers of SCCs and a review into HNC found that 32% showed p53 mutations [37]. CDKN2A has been shown to be commonly altered in OCSCCs, particularly more frequently in HPV negative tumors, compared with HPV positive tumors [36]. TP53 is a key tumor suppressor gene, known to be significantly affected in many squamous cell carcinomas. Heterozygous or even homozygous mutations in the p53 gene lead to aberrant function of the p53 protein, contributing as a permissive factor of oncogenesis. Our study validates research work attempting to understand and control TP53 mutations, as it remains a key driver of oncogenesis in the oral cavity. If a future therapeutic were to be developed that controlled TP53 mutations, it would be reasonable to expect a significance response in SCCs from the oral cavity.

The differences between smoking status groups were of interest to this study. Participants who had ever smoked (current and ex-smokers combined), compared with the non-smoking population displayed gene expression changes positive correlated with increased TP53 transcriptional regulation and mTOR signaling and negatively correlated with mitochondrial respiration. Some of these associations have been previously explored in head and neck carcinomas and other cancers. mTOR pathway has been linked to multiple smoking-related cancers including: laryngeal SCC, small cell lung carcinoma and transitional cell bladder carcinoma [38–42]. Work performed in lung carcinoma has shown the ability of extracts from cigarette smoke to drive activators of the mTOR pathway [41]. Additionally, in a study on laryngeal SCC, a cancer which is strongly correlated with tobacco exposure, mTOR and EGFR pathways were highly overexpressed. However, no study was identified which found links between the mTOR pathway and compared the effects between smoking and non-smoking groups, making a comparison with this study difficult. Overall, the up-regulation in mTOR pathways exclusively in the smoking population may indicate that there are genetically different drivers in the smoking population, compared with non-smokers.

## Conclusions

This research highlights a number of key understandings of the etiology of oral cavity cancer. Firstly, OCSCC is a disease which is not commonly due to HPV, with HPV presence and induced changes see in less than 5% of the OCSCC samples. Utilizing exome sequencing this study determined that the most significant mutation amongst the participants in this study was in the *TP53* gene. This study has found that changes in TP53 transcriptional regulation, mTOR signaling and mitochondrial function are more common-place in the smoking population. This knowledge may prove useful for further research investigating therapeutic options in these patients. Finally, we note that OCSCC is a highly complex and heterogeneous disease that requires further research as it is still currently ill-characterized despite the availability or large-scale genomics projects like the TCGA project. This may be potentially achieved involving large multicenter designed trials in order to understand the intricacies of the biology of OCSCC.

## Supporting information

S1 Fig Normalized read counts of HPV16 E7 gene. Reads from RNA-seq data (33 samples) were aligned to the human reference genome concatenated with HPV16 (NC_001526.4) genome and represented as normalized read counts per million for the HPV16 E7 oncogene.
(EPS)

S2 Fig Positive immunohistochemical stain for p16 in tumor sample. P16 immunohistochemical staining was performed on 27 tumor samples. Six of these samples were considered p16 positive by pathology, with only a single tumor sample demonstrating positive p16 immunohistochemical staining and HPV16 mRNA positivity.
(EPS)

S3 Fig Waterfall plot of significant mutations identified by MuTect, MuTect2 or VarScan2. Mutations are ranked accordingly to significance assigned by MutSigCV analysis. Clinical traits are matched to the samples and displayed as a heat map. Tumor purity and ploidy values predicted from BubbleTree are included.
(EPS)

S4 Fig BubbleTree Tumor purity estimates for samples 1–10. The BubbleTree graph is presented with the R score (copy number ratio) on the x-axis and the HDS (heterozygous-deviation score; |Beta allele frequency– 0.5|) on the y-axis. The proximity of the bubbles (somatic copy number alterations), or leaves, to the tree branches indicates the integer allele-specific copy numbers. Tumor purity is determined by the somatic copy number alterations segments at the highest HDS values.
(EPS)

S5 Fig BubbleTree Tumor purity estimates for samples 11–20. The BubbleTree graph is presented with the R score (copy number ratio) on the x-axis and the HDS (heterozygous-deviation score; |Beta allele frequency– 0.5|) on the y-axis. The proximity of the bubbles (somatic copy number alterations), or leaves, to the tree branches indicates the integer allele-specific copy numbers. Tumor purity is determined by the somatic copy number alterations segments at the highest HDS values.
(EPS)

S6 Fig BubbleTree Tumor purity estimates for samples 21–25. The BubbleTree graph is presented with the R score (copy number ratio) on the x-axis and the HDS (heterozygous-deviation score; |Beta allele frequency– 0.5|) on the y-axis. The proximity of the bubbles (somatic copy number alterations), or leaves, to the tree branches indicates the integer allele-specific copy numbers. Tumor purity is determined by the somatic copy number alterations segments at the highest HDS values.
(EPS)

S7 Fig Differential gene testing between smoking groups. Differential gene expression testing between the smoking groups (Current versus Ex; Current versus Never; Ex vs Never) using LIMMA-voom. Genes were considered statistically significanct if they met the fold-change threshold of > 1.25 or < -1.25 and Benjamini and Hochberg (BH) adjusted p-value of < 0.05. The result is shown as a VennDiagram.
(EPS)

S8 Fig Consensus WGCNA network analysis between TCGA HNSC-OCSCC and BNE PAH OCSCC RNA-seq data. (A) Module preservation z-score of modules detected in consensus gene network. Higher z-score indicates higher module preservation/similarity (>10 z-score). (B) Correlation of modules detected in consensus gene network with clinical trait measurements is shown as a module-trait table where the correlation score is presented from a range of -1 to 1 (green to white to red). Grey boxes indicates lack of consensus between the two datasets.
(EPS)
